# Supplementary material for: Live-imaging of endothelial Erk activity reveals dynamic and sequential signalling events during regenerative angiogenesis
Source: eLife. 2021 May 18;10:e62196. doi: 10.7554/eLife.62196 (PMC8175085; doi:10.7554/eLife.62196)
Supplement: Transparent reporting form [file elife-62196-transrepform.docx]

***eLife’s* transparent reporting form**

We encourage authors to provide detailed information *within their submission* to facilitate the interpretation and replication of experiments. Authors can upload supporting documentation to indicate the use of appropriate reporting guidelines for health-related research (see [EQUATOR Network](http://www.equator-network.org/%20)), life science research (see the [BioSharing Information Resource](https://biosharing.org/)), or the [ARRIVE guidelines](http://www.plosbiology.org/article/info:doi/10.1371/journal.pbio.1000412) for reporting work involving animal research. Where applicable, authors should refer to any relevant reporting standards documents in this form.

If you have any questions, please consult our Journal Policies and/or contact us: [editorial@elifesciences.org](mailto:editorial@elifesciences.org).

**Sample-size estimation**

- You should state whether an appropriate sample size was computed when the study was being designed
- You should state the statistical method of sample size computation and any required assumptions
- If no explicit power analysis was used, you should describe how you decided what sample (replicate) size (number) to use

Please outline where this information can be found within the submission (e.g., sections or figure legends), or explain why this information doesn’t apply to your submission:

Power calculations were not used.

Individual embryos were considered as replicates throughout the study and sufficient numbers of embryos used to provide strong statistical significance in each experiment. All data is presented with individual data points indicated so that n-values and replicates are clearly stated and transparent.
Due to the use of zebrafish embryos at early stages, large numbers of embryos were used throughout with no ethical requirements to limit embryo numbers.

**Replicates**

- You should report how often each experiment was performed
- You should include a definition of biological versus technical replication
- The data obtained should be provided and sufficient information should be provided to indicate the number of independent biological and/or technical replicates
- If you encountered any outliers, you should describe how these were handled
- Criteria for exclusion/inclusion of data should be clearly stated
- High-throughput sequence data should be uploaded before submission, with a private link for reviewers provided (these are available from both GEO and ArrayExpress)

Please outline where this information can be found within the submission (e.g., sections or figure legends), or explain why this information doesn’t apply to your submission:

Individual embryos were considered as biological replicates throughout the study and sufficient numbers of embryos used to provide statistical significance in each experiment.

All data is presented with individual data points indicated throughout the paper so that n-values (cells or embryos) and replicates (embryos) are clearly stated and transparent.
No data was excluded and no outliers removed.

**Statistical reporting**

- Statistical analysis methods should be described and justified
- Raw data should be presented in figures whenever informative to do so (typically when N per group is less than 10)
- For each experiment, you should identify the statistical tests used, exact values of N, definitions of center, methods of multiple test correction, and dispersion and precision measures (e.g., mean, median, SD, SEM, confidence intervals; and, for the major substantive results, a measure of effect size (e.g., Pearson's r, Cohen's d)
- Report exact p-values wherever possible alongside the summary statistics and 95% confidence intervals. These should be reported for all key questions and not only when the p-value is less than 0.05.

Please outline where this information can be found within the submission (e.g., sections or figure legends), or explain why this information doesn’t apply to your submission:

Statistical analysis methods are transparently indicated for all experiments in the text, figure legends and methods. This includes the tools used, statistical methods used, p-values and what is indicated on the graphs (eg. mean, median, SD).

Sample analysis tools and approaches used to quantify fluorescence intensity are clearly indicated in the methods.

P-values are indicated as precise values (unless p<0.00001) in the Figures for the key experimental outcomes both significant and non-significant.

(For large datasets, or papers with a very large number of statistical tests, you may upload a single table file with tests, Ns, etc., with reference to sections in the manuscript.)

**Group allocation**

- Indicate how samples were allocated into experimental groups (in the case of clinical studies, please specify allocation to treatment method); if randomization was used, please also state if restricted randomization was applied
- Indicate if masking was used during group allocation, data collection and/or data analysis

Please outline where this information can be found within the submission (e.g., sections or figure legends), or explain why this information doesn’t apply to your submission:

All quantification of treatments in experimental groups was done blind using software as described in the methods to ensure no investigator bias.

Analysis of data values and statistics were only performed following such unbiased analysis of experimental data once groups were complete.

**Additional data files (“source data”)**

- We encourage you to upload relevant additional data files, such as numerical data that are represented as a graph in a figure, or as a summary table
- Where provided, these should be in the most useful format, and they can be uploaded as “Source data” files linked to a main figure or table
- Include model definition files including the full list of parameters used
- Include code used for data analysis (e.g., R, MatLab)
- Avoid stating that data files are “available upon request”

Please indicate the figures or tables for which source data files have been provided:

Source data for image analysis is now included as raw values in a master excel sheet for all measurements used in every Figure panel.
Throughout the manuscript we directly display individual tracks and datapoints for each measurement made and so provide all specific measurements in this source data file.
